# An explorative metabolomic analysis of the endothelium in pulmonary hypertension

**DOI:** 10.1038/s41598-022-17374-x

**Published:** 2022-08-02

**Authors:** J. Carlsen, H. H. Henriksen, I. Marin de Mas, P. I. Johansson

**Affiliations:** 1 Department of Cardiology, 2141 Copenhagen University Hospital, Rigshospitalet, Blegdamsvej 9, 2100, Copenhagen, Denmark; 2 Department of Clinical Immunology, Copenhagen University Hospital, Rigshospitalet, Copenhagen, Denmark; 3 CAG Center for Endotheliomics, Copenhagen University Hospital, Rigshospitalet, Copenhagen, Denmark; 4 Novo Nordisk Foundation Center for Biosustainability, Danish Technical University, Lyngby, Denmark

**Keywords:** Cardiology, Molecular medicine

## Abstract

Pulmonary hypertension (PH) is classified into five clinical diagnostic groups, including group 1 [idiopathic pulmonary arterial hypertension (IPAH) and connective tissue disease-associated PAH (CTD-aPAH)] and group 4 (chronic thromboembolic pulmonary hypertension (CTEPH)). PH is a progressive, life-threatening, incurable disease. The pathological mechanisms underlying PH remain elusive; recent evidence has revealed that abnormal metabolic activities in the endothelium may play a crucial role. This research introduces a novel approach for studying PH endothelial function, building on the genome-scale metabolic reconstruction of the endothelial cell (EC) to investigate intracellular metabolism. We demonstrate that the intracellular metabolic activities of ECs in PH patients cluster into four phenotypes independent of the PH diagnosis. Notably, the disease severity differs significantly between the metabolic phenotypes, suggesting their clinical relevance. The significant metabolic differences between the PH phenotypes indicate that they may require different therapeutic interventions. In addition, diagnostic capabilities enabling their identification is warranted to investigate whether this opens a novel avenue of precision medicine.

## Introduction

Pulmonary hypertension (PH) is a progressive disease with no available curative therapy, and despite recent advances in pharmacological therapy, the 5-year survival remains as low as 65–70^1,2^. Plasma metabolomic studies in idiopathic PAH (IPAH) have demonstrated that alterations in glucose homeostasis, lipid metabolism, and altered bioenergetics are associated with worse outcomes^3,4^. Metabolomic analysis of bone morphogenetic protein receptor type 2 mutations in the pulmonary endothelium revealed widespread metabolic reprogramming^5^, and recently, a phase 2 trial demonstrated that sotatercept, which targets dysfunctional bone morphogenetic protein pathway signalling, was efficacious in treating PAH^6^. The plasma metabolomic profile in chronic thromboembolic pulmonary hypertension (CTEPH) suggests aberrant lipid metabolism characterised by increased lipolysis, fatty acid oxidation, and ketogenesis^7^.

The endothelium is under normal conditions in a quiescent state. When activated, the endothelium secretes different growth factors and cytokines that affect endothelial cell (EC) proliferation, apoptosis, and coagulation; attract inflammatory cells; and/or affect vasoactivity to restore homeostasis^8^. Chronic activation of the endothelium leads to EC dysfunction, resulting in pathological changes, such as those observed in PH^9,10^. Many factors have been suggested to trigger EC dysfunction in PH, such as shear stress, hypoxia, inflammation, cilia length, and genetics^11–13^. The overactivated endothelium in PH secretes vasoconstrictive^14,15^ and proliferative factors^15–17^ and reduces the secretion of vasodilators, which indicates that EC dysfunction might play a central role in the pathogenesis of PH.

This study introduces a novel approach for investigating intracellular metabolism using a genome-scale metabolic reconstruction of the microvascular EC^18^. This EC model is constructed from the metabolic models of humans (RECON1), metabolomics data from human umbilical vein endothelial cells, a manual literature review of endothelial metabolism, and finally, transcriptomic data from three subtypes of endothelial cells (human umbilical vein, human mammary vascular, and human pulmonary artery) making it relevant for investigating the PH population^19,20^.

## Results

### Clinical characteristics of PH patients

All patients were prevalent and were differently treated according to clinical groups PAH, CTD-aPAH and CTEPH in agreement with international guidelines^1^. PAH patients were treated with double- or triple therapy (n = 1/n = 11), CTD-aPAH mono- or double treatment (n = 6/n = 6) and CTEPH no-, mono- or double treatment (n = 2, n = 5 and n = 1). The duration of disease in the PAH group was 71 months (IQR 30, 99), CTD-aPAH 39 months (IQR 18, 96), and CTEPH 49 months (IQR 13, 169).

The clinical characteristics of patients with each PH subtype and catecholamine measurements are presented in Table 1. The median age was 58 (IQR 44, 68) for PH patients and 40 (IQR 33, 51) for controls. The PH patients consisted of 51% males and the controls of 70% males.

**Table 1:** Patient demographics, admission vitals, disease severity, and catecholamine measurements in 35 PH patients. Medians (IQR) or n (%) are reported.

		IPAH	CTD-aPAH	CTEPH
		(n = 12)	(n = 12)	(n = 11)
**Demography**				
Age	Years	48.0 [39.8, 60.3]	64.0 [47.0, 74.3]	61.0 [51.5, 66.0]
Sex	Male, n (%)	9 (75.0%)	2 (16.7%)	7 (63.6%)
**Echocardiography**				
TR	mmHg	62.0 [48.3, 77.0]	54.5 [47.8, 59.5]	60.0 [50.0, 65.0]
**Right heart catheterisation**				
mPAP	mmHg	54.0 [52.0, 63.0]	45.5 [29.8, 51.3]	42.0 [34.3, 48.0]
CI	L/min/m^2^	1.60 [1.25, 2.12]	2.90 [2.38, 3.25]	3.10 [2.90, 3.60]
PCWP	mmHg	12.0 [11.0, 13.5]	10.5 [7.75, 14.3]	11.0 [10.0, 14.0]
PVR	Wood units	13.5 [12.0, 15.6]	6.45 [4.38, 9.75]	5.20 [4.43, 6.38]
**Laboratory parameter**				
NT-proBNP	% max ULN	271 [265, 919]	158 [99.2, 745]	148 [94.6, 420]
**ELISA measurements**				
Epinephrine	pg/mL	14.4 [6.15, 25.0]	14.0 [6.44, 31.1]	16.9 [8.03, 27.5]
Norepinephrine	pg/mL	236 [139, 425]	386 [227, 461]	458 [213, 578]
**Outcome**				
6-min walking distance	m	539 [473, 595]	410 [345, 494]	490 [416, 543]
**WHO functional class**				
I	Yes n (%)	1 (8.3%)	0 (0%)	2 (18.2%)
II	Yes n (%)	3 (25.0%)	6 (50.0%)	8 (72.7%)
III	Yes n (%)	7 (58.3%)	6 (50.0%)	1 (9.1%)
IV	Yes n (%)	1 (8.3%)	0 (0%)	0 (0%)

CI, cardiac index; mPAP, mean pulmonary arterial pressure; NT-proBNP, N-terminal (*NT*)-pro brain natriuretic peptide (BNP); PCWP, pulmonary capillary wedge pressure; PVR, pulmonary vascular resistance; TR, tricuspid regurgitation; ULN: upper limit of normal.

### Plasma metabolite concentrations differ between healthy controls and PH patients

**Figure 1:**
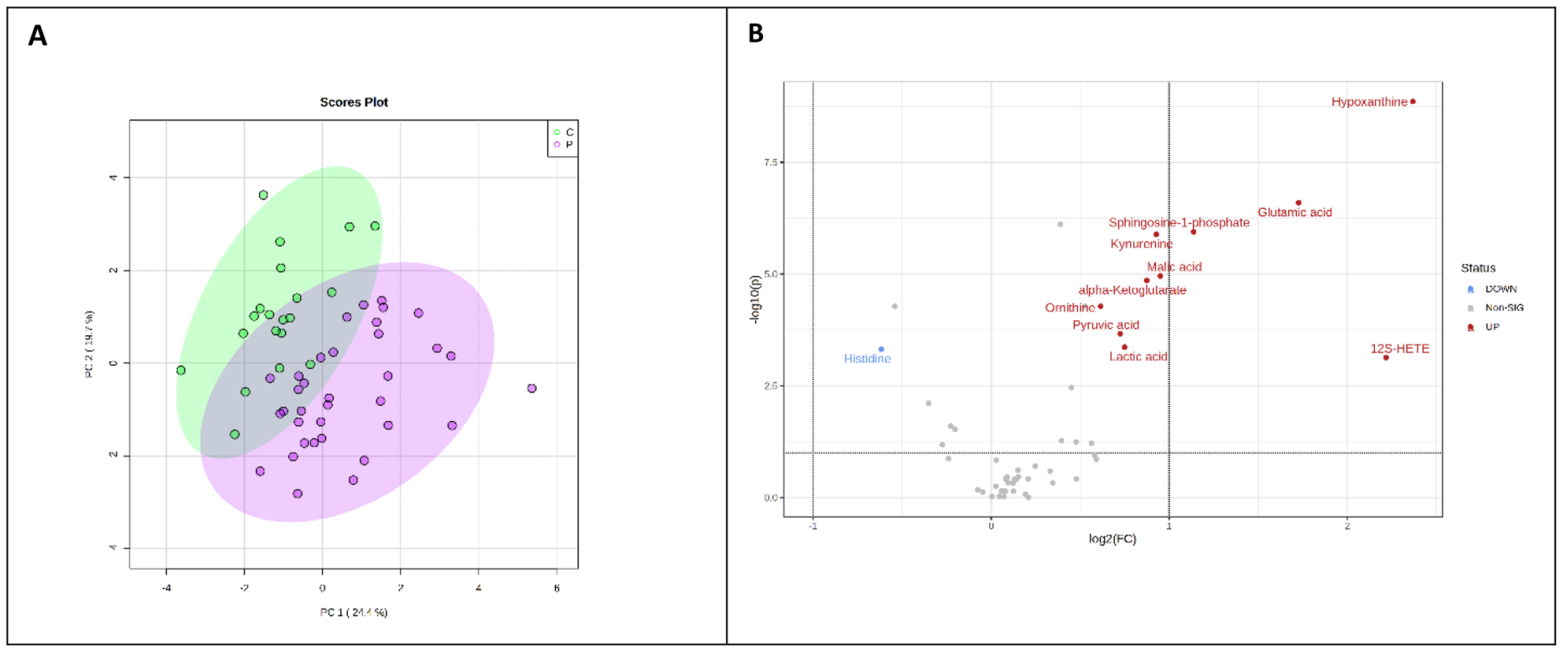
Plasma metabolite concentrations differ between healthy controls and PH patients. (**A**) Principal component analysis was performed between patients and controls. (**B**) A Vulcano plot identifies metabolites that differ among the patients compared to the healthy controls with a 1.5 fold-change and FDR corrected. The direction of comparison was patients/controls.

Principal component analysis was performed between patients and controls; the first two principal components (PCs) explained 44.1% of the total variance (Fig. 1A). A Vulcano plot identifies metabolites that differ among the patients compared to healthy controls (Fig. 1B).

### Intracellular metabolic activities analysis

A complete overview of the results from the examined cellular activities are appended in Supplement Material A. Nineteen cellular activities had constant values of either 0 flux or 1000 flux indicating inactive or fully activated pathway across the samples, and were removed from further analysis.

**Figure 2:**
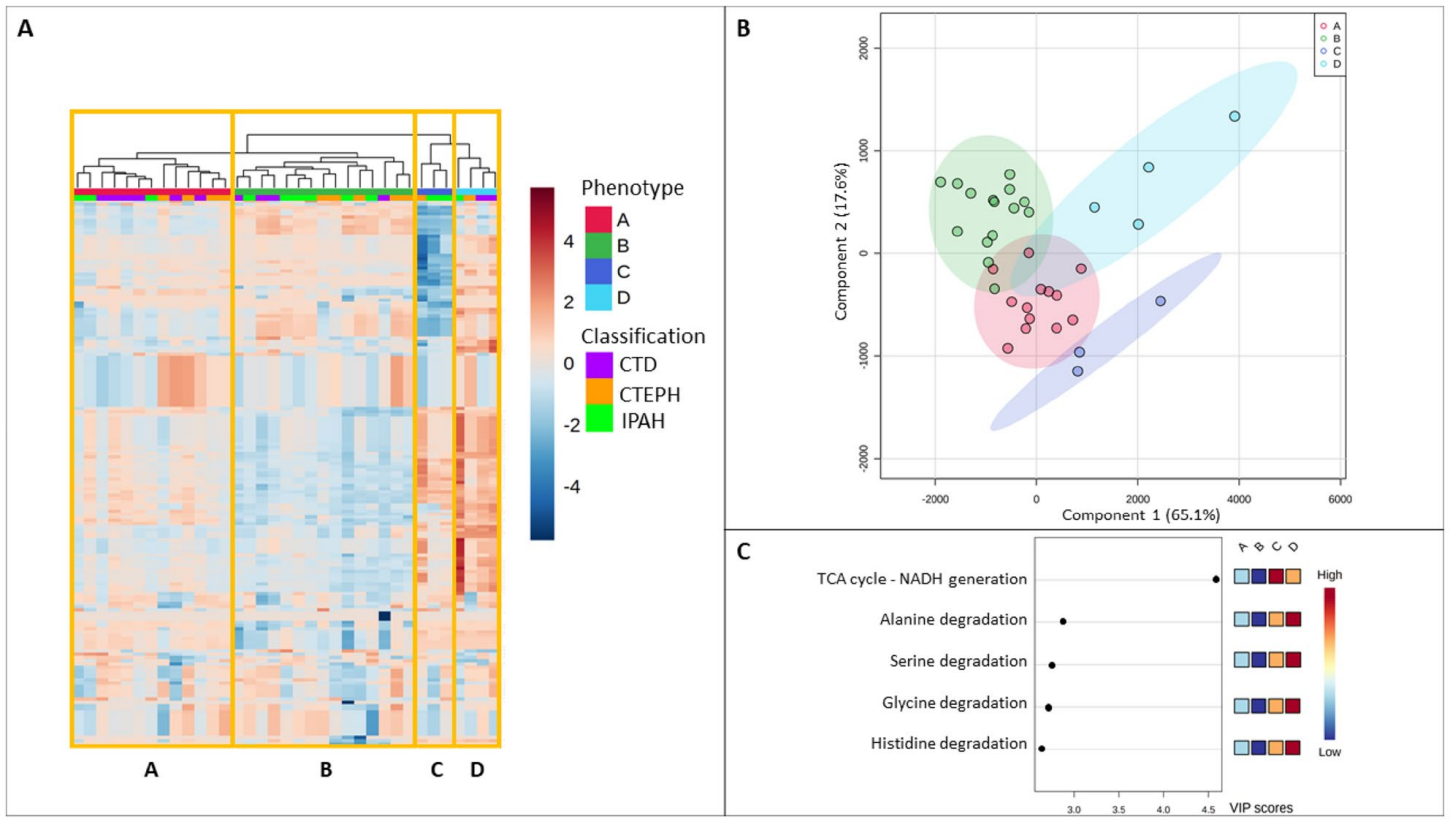
Four distinct intracellular EC metabolic clusters in patients with PH. (**A**) Heatmap displays EC intracellular metabolic activities, analysed using iEC3006, in 35 patients with PH. (**B**) Partial Least-Squares Discriminant Analysis, PLS-DA, identified different intracellular activities among PH phenotypes. (**C**) Top 5 list of variables of importance in the PLS-DA.

When investigating the PH patients’ profiles on the basis of cellular metabolic activities, four distinct clusters (Phenotypes A–D) were revealed that were independent of clinical PH diagnosis (Fig. 2A). PLS-DA analysis revealed that the top 20 metabolic cellular activities separating the four phenotypes were NADH generation (VIP score above 4.5) followed by the degradation of 19 amino acids (Fig. 2B,C, Supplementary Material B).

### Clinical characteristics of the metabolic cellular activities of phenotypes A–D

The clinical characteristics and catecholamine measurement of patients with each phenotype based on metabolic cellular activities activity are presented in Table 2. Phenotype C had a median fourfold higher level of NT-proBNP than phenotype A, and phenotype D had a 14-fold higher level of NT-proBNP than phenotype B; phenotypes C and D also had a higher proportion of patients with WHO functional classifications III and IV.

**Table 2:** Clinical characteristics and catecholamine measurements of PH phenotypes A–D. Medians (IQR) or n (%) are reported.

		A	B	C	D
		**(n = 13)**	**(n = 15)**	**(n = 3)**	**(n = 4)**
**PH classification**					
IPAH	Yes n (%)	3 (23.1%)	6 (40.0%)	2 (66.7%)	1 (25.0%)
CTD-aPAH	Yes n (%)	6 (46.2%)	4 (26.7%)	0 (0%)	2 (50.0%)
CTEPH	Yes n (%)	4 (30.8%)	5 (33.3%)	1 (33.3%)	1 (25.0%)
**Demography**					
Age	Years	56.0 [47.0, 61.0]	54.0 [41.5, 71.0]	63.0 [53.5, 64.0]	67.0 [56.0, 74.3]
Sex	Male n (%)	6 (46.2%)	7 (46.7%)	3 (100%)	2 (50.0%)
**Echocardiography**					
TR	mmHg	53.5 [49.3, 68.8]	57.0 [44.3, 73.8]	57.0 [43.5, 63.5]	62.0 [59.3, 64.5]
**Right heart catheterisation**					
mPAP	mmHg	50.0 [47.3, 60.5]	40.0 [30.0, 47.8]	60.0 [56.5, 62.5]	49.5 [45.8, 52.5]
CI	L/min/m^2^	2.35 [2.15, 2.68]	3.20 [1.60, 3.60]	2.10 [2.03, 2.45]	2.90 [2.30, 3.23]
PCWP	mmHg	11.0 [9.50, 15.0]	10.0 [8.75, 14.0]	12.0 [11.0, 12.0]	13.0 [11.8, 14.0]
PVR	Wood units	10.0 [6.35, 12.8]	5.40 [4.13, 10.5]	12.0 [10.0, 12.5]	7.95 [6.25, 12.0]
**Laboratory parameter**					
NT-proBNP	% max ULN	208 [98.9, 517]	141 [87.2, 265]	809 [637, 864]	1980 [1710, 2210]
**ELISA measurements**					
Epinephrine	pg/mL	15.3 [6.54, 27.2]	10.9 [6.15, 25.0]	16.7 [15.1, 22.1]	26.1 [19.3, 45.9]
Norepinephrine	pg/mL	358 [170, 477]	328 [159, 428]	496 [260, 864]	644 [352, 964]
**Outcome**					
Walking distance	Yes n (%)	426 [394, 504]	552 [475, 614]	410 [325, 481]	443 [379, 498]
**WHO functional class**					
I	Yes n (%)	1 (7.7%)	2 (13.3%)	0 (0%)	0 (0%)
II	Yes n (%)	7 (53.8%)	9 (60.0%)	0 (0%)	1 (25.0%)
III	Yes n (%)	5 (38.5%)	4 (26.7%)	3 (100%)	2 (50.0%)
IV	Yes n (%)	0 (0%)	0 (0%)	0 (0%)	1 (25.0%)

CI, cardiac index; CTD-aPAH, connective tissue disease associated PAH; CTEPH, chronic thromboembolic pulmonary hypertension; IPAH, idiopathic pulmonary arterial hypertension; mPAP, mean pulmonary arterial pressure; NT-proBNP, N-terminal (*NT*)-pro brain natriuretic peptide (BNP) ; PCWP, pulmonary capillary wedge pressure; PVR, pulmonary vascular resistance; TR, tricuspid regurgitation; ULN: upper limit of normal.

### Comparison of cellular metabolic pathway activities in phenotypes A–D

#### Amino acid metabolism

Compared with phenotypes A and B, phenotype D had significantly increased degradation of amino acids (alanine, glycine, serine, histidine, asparagine, phenylalanine, valine, proline, tyrosine, aspartate, threonine, isoleucine, tryptophan, glutamate, leucine, glutamine, cysteine, ornithine, and lysine), indicative of increased catabolism; phenotype C had significantly increased degradation of amino acids expect for ornithine and lysine compared with phenotype B (Supplementary Material C).

#### Energy metabolism

**Figure 3:**
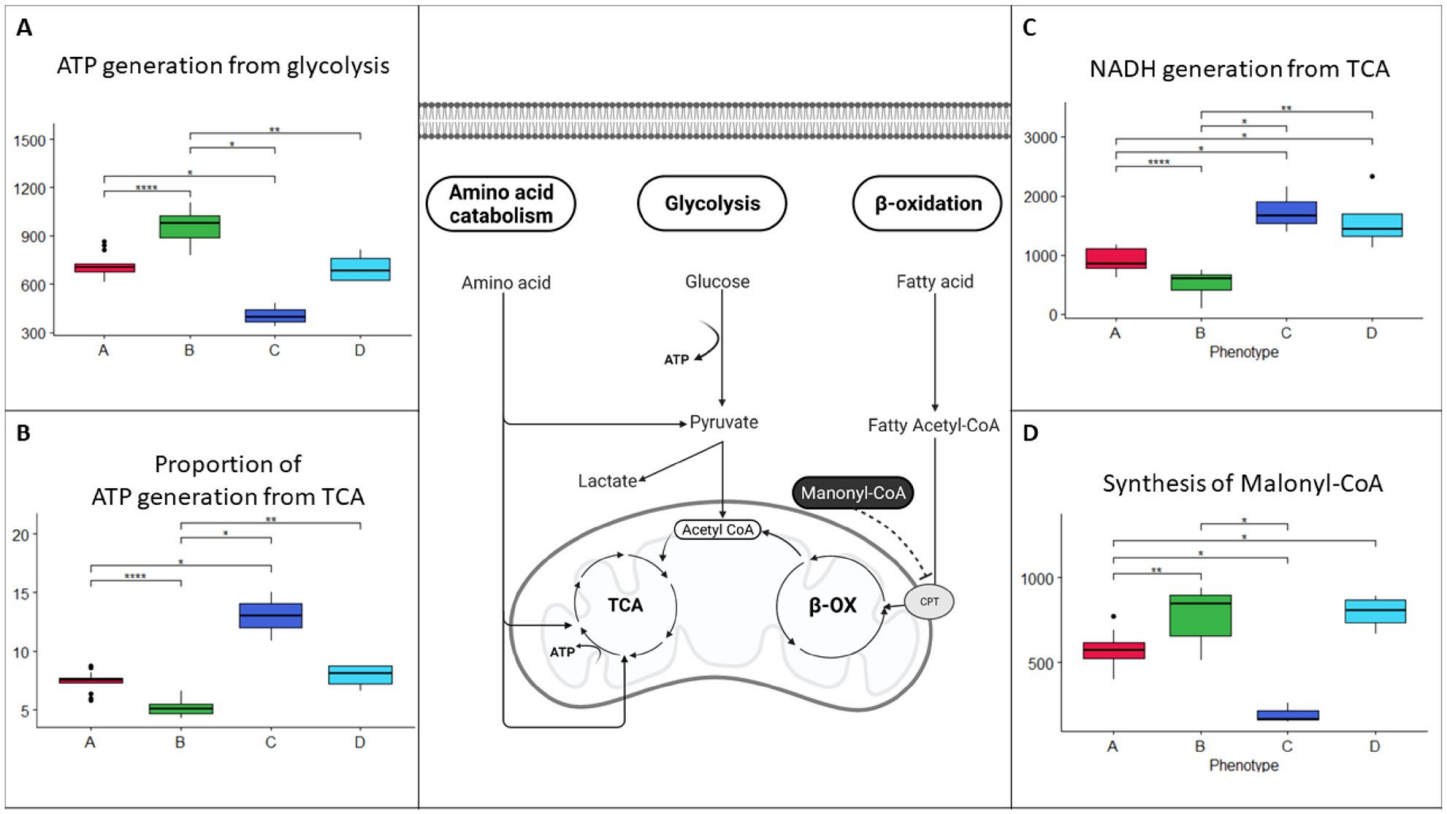
Metabolic cellular activities in phenotypes (**A**–**D**)**. **(**A**) ATP generation from glycolysis in the four PH phenotypes. (**B**) Proportion of ATP generation from TCA (overall ATP generation from glycolysis and TCA/ATP generation from glycolysis) in the four PH phenotypes. (**C**) NADH generation by TCA in the four PH phenotypes. (**D**) Synthesis of malonyl-CoA in the four phenotypes. The cellular overview was created with BioRender.com.

Phenotype B had the highest ATP generation from glucose, and phenotype C had lower ATP generation from glucose than phenotype A (Fig. 3A). Phenotype C had the highest and phenotype B had the lowest proportion of ATP generation from the tricarboxylic acid (TCA) cycle (Fig. 3B).

#### TCA metabolism

Phenotype B had the lowest NADH generation by the TCA cycle, whereas phenotypes C and D had the highest (Fig. 3C).

#### Lipid metabolism

Phenotype C had lower activity in malonyl-CoA synthesis than phenotypes A and B, and phenotype A had lower activity than phenotypes B and C (Fig. 3D).

## Discussion

The main finding of the present investigation was that the intracellular metabolic activity of ECs of PH patients clustered into four phenotypes (A–D) that were independent of the PH diagnosis. Notably, the disease severity (reflected by NT-proBNP levels) differed significantly between the metabolic phenotypes, suggesting their clinical relevance^21^; phenotype D had the highest levels, and phenotype B had the lowest levels.

Our observations are in alignment with those of Kariotis and colleagues, who identified three whole-blood transcriptomic patient subgroups of IPAH patients that accounted for more than 90% of the cohorts^22^. These subgroups were associated with poor, moderate, and good prognoses.. Our study also extends to CTEPH patients, suggesting that the metabolic phenotypes are not restricted to PAH. Because the present study is, to the best of our knowledge, the first to explore the inferred intracellular endothelial metabolism in PH patients in the context of a genome-scale metabolic model (GEM), making direct comparisons with the literature is difficult. EC research in PH largely builds on cell culture experiments, which use ECs from PH patients undergoing lung transplantation or from biorepositories^23^. Unfortunately, these studies have been conducted under static conditions without hemodynamic shear stress, and the manipulated cells have been cultured on plastic, which is five orders of magnitude stiffer than in vivo conditions^23^; thus, a direct comparison with results from metabolic systems analysis is difficult. Similarly, PH animal models poorly mimic complex human disease^24^ and require extensive manipulation of the cells. This may explain the lack of previous reports on the identification of clinically relevant EC phenotypes with distinctly different intracellular metabolic activities.

In our exploratory modelling analyses, phenotype B had the lowest overall ATP generation but displayed the highest ATP generation from glycolysis. Prioritising glycolysis over oxidative phosphorylation may enable ECs to increase lactate production and thus promote angiogenesis^25–28^, which has previously been identified as a vital feature in studies of general metabolism in PAH vascular cells^29,30^. Utilising the glycolytic path may also minimise reactive oxygen species (ROS) production produced by oxidative phosphorylation in the TCA cycle and generate ATP more rapidly than the TCA cycle. Similar metabolic reprogramming has also been reported in cancer, providing ECs with a survival advantage^31^. The finding that phenotype B had the lowest NT-proBNP level and thus the mildest disease severity in the PH patients included in this study suggests that these ECs are subject to the least cellular stress.

Phenotype D had the highest disease severity as measured by NT-proBNP, which may be a consequence of high cellular stress, and thereby, elevated intracellular reactive oxygen species force the cell to increase amino acid degradation^32^. This is consistent with our findings in this study that amino acid degradation, particularly increased in phenotype D, was 19 of the top 20 pathways to distinct the phenotypes. Also, phenotype D had the highest levels of circulating catecholamines. In alignment with this, neurohumoral signalling, including sympathetic activation, was recently reported to contribute to the PAH pathophenotype^33^. Furthermore, Nagaya and colleagues investigated 60 PH patients and reported that plasma norepinephrine was significantly increased in functional class IV compared with functional classes II and III^34^. Conversely, in phenotype A, which exhibited severalfold lower NT-proBNP levels than phenotype D, significantly lower amino acid degradation and higher β-oxidation were observed.

In addition, phenotype C, which had the second-highest disease severity as measured by NT-proBNP levels, displayed inverse ATP generation compared with phenotype B, with the lowest ATP generation from glycolysis and the highest oxidative phosphorylation, providing fuel to the TCA cycle from high amino acid degradation and beta-oxidation of fatty acids induced by low synthesis of malonyl-CoA.. Whether the differences in intracellular metabolic activities of ECs between the different PH phenotypes reflect differences in disease severities or genetic variation remain to be investigated.

The present study has several limitations inherent to its observational design; thus, no causal effect can be inferred. In addition, a limited number of PH patients from a single centre were studied, and this may further limit the generalisability of the results. Importantly, the PH patients received various pharmacological therapies with agents potentially affecting the endothelium, and the observed metabolic perturbations may be influenced by this. However, sample sizes will have to be considered, and validation of treatment impact on phenotype will await larger multicenter studies. Although the iEC3006 is the most comprehensive metabolic reconstruction of the EC to date, expansion of the metabolic coverage focusing on membrane lipids and glycan metabolism is required. Furthermore, the exploratory modeling analyses were based on a limited number of extracellular metabolites in the plasma, which may affect the intracellular metabolic cellular activity analysis despite covering all parts of the central metabolism^35^. Consequently, the inferred intracellular pathways analyses need to be validated in vitro under physiological settings. Also, metabolites from other cell types in plasma cannot, at the moment, be incorporated in the iEC3006 since it is a genome-scale single-cell metabolic model. However, our approach that plasma metabolism primarily reflects the EC alteration is based on the fact that 1 trillion EC is in constant contact with the circulating blood. Lastly, since SNP data were unavailable, we could not evaluate if the phenotype may reflect a continuum of PH progression severity or genetic differences.

## Conclusions

The present exploratory study suggests that the endothelium displays four distinctly different intracellular metabolic phenotypes independent of the patient’s PH diagnosis. The phenotypes are characterised by differences in ATP generation associated with varying disease severity as evaluated by NT-proBNP. The substantial metabolic differences between the PH phenotypes suggest that they may require different therapeutic interventions; furthermore, diagnostic capabilities enabling their identification is warranted to investigate whether this opens a novel avenue of precision medicine^36^.

## Methods

### Setting and patients

The cohort included adult (≥ 18 years) PH patients admitted to the Department of Cardiology, Copenhagen University Hospital, Rigshospitalet, Copenhagen, Denmark. The study was approved by the Ethics Committees in the Capital Region of Denmark (H-17021130) and was conducted in line with the Declaration of Helsinki. Blood samples were collected from a peripheral vessel upon arrival at the ambulatory clinic, and informed consent was obtained from all patients.

### Patient selection

Patients were randomly selected retrospectively for enrollment in this study on the basis of their PH diagnosis from a biorepository of over 450 patients. All patients had the PH diagnosis confirmed with right heart catheterizations, including measurements of pulmonary pressures and cardiac output/index using the thermodilution method. In total, 12 patients with IPAH, 12 patients with CTD-aPAH, and 11 patients with CTEPH were selected, along with 20 healthy volunteers that have previously been published^18^. Clinical data from the patients were retrieved from a research database and electronic health records.

### Analysis of clinical characteristics

Statistical analysis was performed using Rstudio (version 3.6.3). Patients’ descriptive data are presented as medians with interquartile ranges (IQRs) or as percentages (%). The nonparametric Kruskal–Willis test was used to evaluate differences in the main cellular metabolic activities in the four metabolic phenotypes.

NT-proBNP was adjusted to the standard maximum reference value as a percentage (i.e. the maximum reference value was equal to 100%).

### Sample Preparation

Blood samples were collected in EDTA tubes. Tubes were immediately centrifugated at 1,800 × *g* for 10 min at 5 °C two times to separate plasma. Plasma was aliquoted and frozen at − 80 °C.

### Enzyme-linked immunosorbent assay (ELISA) measurement

Epinephrine and norepinephrine were measured according to the manufacturer’s recommendations (2-CAT ELISA, Labor Diagnostica Nord GmbH Co. & KG, Nordhorn, Germany).

### Mass spectrometry analysis

Ultra-high performance liquid chromatography–mass spectrometry (UHPLC-MS) analysis was performed as previously published^37^ using the Vanquish UHPLC system (Thermo Fisher Scientific, San Jose, CA, USA) with a Q Exactive mass spectrometer (HF Hybrid Quadrupole-Orbitrap, Thermo Fisher Scientific, San Jose, CA, USA). An electrospray ionisation interface was used as an ionisation source. The analysis was performed in negative and positive ionisation modes. A QC sample was analysed in MS/MS mode for the identification of compounds. UPLC was performed using the protocol described by Catalin et al., slightly modified so that the derivatisation reaction was stopped by the addition of chloroform ^38^. Peak areas were extracted using Compound Discoverer 2.0 (ThermoFischer Scientific, Waltham, MA, USA).

Gas chromatography–mass spectrometry (GC–MS) was used to detect amino and non-amino organic acids (7890B, Agilent, Santa Clara, CA, USA) coupled with a quadrupole mass spectrometry detector (5977B, Agient). The system was controlled by ChemStation (Agilent). Raw data were converted to netCDF format using Chemstation (Agilent) before the data was imported and processed in Matlab R2018b (Mathworks, Natick, MA, USA) using the PARADISe software^39^.

### Analysis of mass spectrometry data

On the basis of our prior work in critically ill patients and the ability to integrate the metabolites into the iEC3006, we selected 51 metabolites to be quantified (Supplementary Table D)^18^.

Less than 1% of the total values were missing, and these were imputed using the Missforest package^40^ in RStudio (version 3.6.3). The quantified metabolic data were normalized by log10 transformation and Pareto scaling to create Principal Component Analysis (PCA), and the Volcano plot analysis. P-values were FDR adjusted.

### Analysis of data with the iEC3006 genome-scale metabolic model (GEM)

To date, our GEM EC, entitled iEC3006, is the most expansive genome-scale metabolic model of the EC, including 2,035 genes and 3,006 reactions involving a total of 2,114 metabolites^18^. Quantified metabolic data were analysed with iEC3006 using the Cobratoolbox in Matlab R2017b^41^. First, constraints on the uptake and secretion of metabolites were determined by the upper and lower quartiles of their respective transport reaction flux distributions as determined by random sampling flux analysis of the cultured human umbilical vein endothelial cell (HUVEC) constrained version of iEC3006.

We previously determined the normal metabolic variances by including 20 healthy volunteers variances in order to incorporate metabolic patient data into EC-GEM^18^. Mean fold changes (patient phenotype/healthy volunteers) from the plasma metabolomics data set were then applied to the upper and lower quantiles of each transport reaction to define the uptake and secretion in the patient phenotype models; if necessary, reactions were relaxed to obtain a feasible model (Supplementary Table E and Supplementary Table F). In total, 190 manually curated central metabolic cellular activities were investigated (Supplementary Table G)^35^ using the metabolomics package in Rstudio (version 3.6.3). Constant values of metabolic cellular activities measurement were removed from further analysis. The intracellular metabolic similarity was identified by applying a heatmap with a dendrogram. The Euclidian distance and the complete cluster algorithm were used.

## Supplementary Information

Supplementary Information.

## Data Availability

Code to reproduce modeling analysis is available in the GitHub repository https://github.com/HHEN0042/PH

## References

[1] Galie N (2016). 2015 ESC/ERS Guidelines for the diagnosis and treatment of pulmonary hypertension: The Joint Task Force for the Diagnosis and Treatment of Pulmonary Hypertension of the European Society of Cardiology (ESC) and the European Respiratory Society (ERS): Endorsed by: Association for European Paediatric and Congenital Cardiology (AEPC), International Society for Heart and Lung Transplantation (ISHLT). Eur. Heart. J..

[2] Simonneau G (2019). Haemodynamic definitions and updated clinical classification of pulmonary hypertension. Eur. Respir. J..

[3] Zhao Y (2014). Metabolomic heterogeneity of pulmonary arterial hypertension. PLoS ONE.

[4] Rhodes CJ (2017). Plasma metabolomics implicates modified transfer RNAs and altered bioenergetics in the outcomes of pulmonary arterial hypertension. Circulation.

[5] Fessel JP (2012). Metabolomic analysis of bone morphogenetic protein receptor type 2 mutations in human pulmonary endothelium reveals widespread metabolic reprogramming. Pulm. Circ..

[6] Humbert M (2021). Sotatercept for the treatment of pulmonary arterial hypertension. N. Engl. J. Med..

[7] Heresi GA (2020). Plasma metabolomic profile in chronic thromboembolic pulmonary hypertension. Pulm. Circ..

[8] Wong BW, Marsch E, Treps L, Baes M, Carmeliet P (2017). Endothelial cell metabolism in health and disease: impact of hypoxia. Embo. J..

[9] Budhiraja R, Tuder RM, Hassoun PM (2004). Endothelial dysfunction in pulmonary hypertension. Circulation.

[10] Hadi HA, Carr CS, Al Suwaidi J (2005). Endothelial dysfunction: cardiovascular risk factors, therapy, and outcome. Vascul. Health. Risk. Manag..

[11] Humbert M (2008). Endothelial cell dysfunction and cross talk between endothelium and smooth muscle cells in pulmonary arterial hypertension. Vascul. Pharmacol..

[12] Nicod LP (2007). The endothelium and genetics in pulmonary arterial hypertension. Swiss Med. Wkly..

[13] Dummer A (2018). Endothelial dysfunction in pulmonary arterial hypertension: Loss of cilia length regulation upon cytokine stimulation. Pulm. Circ..

[14] Stewart DJ, Levy RD, Cernacek P, Langleben D (1991). Increased plasma endothelin-1 in pulmonary hypertension: Marker or mediator of disease?. Ann. Intern. Med..

[15] Christman BW (1992). An imbalance between the excretion of thromboxane and prostacyclin metabolites in pulmonary hypertension. N. Engl. J. Med..

[16] Tu L (2011). Autocrine fibroblast growth factor-2 signaling contributes to altered endothelial phenotype in pulmonary hypertension. Am. J. Respir. Cell. Mol. Biol..

[17] Dai Z (2018). Endothelial and smooth muscle cell interaction via FoxM1 signaling mediates vascular remodeling and pulmonary hypertension. Am. J. Respir. Crit. Care. Med..

[18] Henriksen HH (2020). Metabolic systems analysis of shock-induced endotheliopathy (SHINE) in trauma: a new research paradigm. Ann. Surg..

[19] McGarrity S, Anuforo Ó, Halldórsson H, Bergmann A, Halldórsson S, Palsson S (2018). Metabolic systems analysis of LPS induced endothelial dysfunction applied to sepsis patient stratification. Sci. Rep..

[20] McGarrity S, Halldorsson H, Palsson S, Johansson PI, Rolfsson O (2016). Understanding the causes and implications of endothelial metabolic variation in cardiovascular disease through genome-scale metabolic modeling. Front. Cardiovasc. Med..

[21] Berghaus TM, Kutsch J, Faul C, von Scheidt W, Schwaiblmair M (2017). The association of N-terminal pro-brain-type natriuretic peptide with hemodynamics and functional capacity in therapy-naive precapillary pulmonary hypertension: results from a cohort study. BMC Pulm. Med..

[22] Kariotis S (2021). Biological heterogeneity in idiopathic pulmonary arterial hypertension identified through unsupervised transcriptomic profiling of whole blood. Nat. Commun..

[23] Wu D, Birukov K (2019). Endothelial cell mechano-metabolomic coupling to disease states in the lung microvasculature. Front. Bioeng. Biotechnol..

[24] Seok J (2013). Genomic responses in mouse models poorly mimic human inflammatory diseases. Proc. Natl. Acad. Sci. USA.

[25] Hunt TK (2007). Aerobically derived lactate stimulates revascularization and tissue repair via redox mechanisms. Antioxid. Redox. Signal..

[26] Ruan GX, Kazlauskas A (2013). Lactate engages receptor tyrosine kinases Axl, Tie2, and vascular endothelial growth factor receptor 2 to activate phosphoinositide 3-kinase/Akt and promote angiogenesis. J. Biol. Chem..

[27] Sonveaux P (2012). Targeting the lactate transporter MCT1 in endothelial cells inhibits lactate-induced HIF-1 activation and tumor angiogenesis. PLoS ONE.

[28] Végran F, Boidot R, Michiels C, Sonveaux P, Feron O (2011). Lactate influx through the endothelial cell monocarboxylate transporter MCT1 supports an NF-κB/IL-8 pathway that drives tumor angiogenesis. Cancer Res..

[29] Paulin R, Michelakis ED (2014). The metabolic theory of pulmonary arterial hypertension. Circ. Res..

[30] Sutendra G, Michelakis ED (2014). The metabolic basis of pulmonary arterial hypertension. Cell Metab..

[31] Verdegem D, Moens S, Stapor P, Carmeliet P (2014). Endothelial cell metabolism: parallels and divergences with cancer cell metabolism. Cancer Metab..

[32] Schieber M, Chandel NS (2014). ROS function in redox signaling and oxidative stress. Curr Biol..

[33] Maron BA, Leopold JA (2015). Emerging concepts in the molecular basis of pulmonary arterial hypertension: Part II: neurohormonal signaling contributes to the pulmonary vascular and right ventricular pathophenotype of pulmonary arterial hypertension. Circulation.

[34] Nagaya N (2000). Plasma brain natriuretic peptide as a prognostic indicator in patients with primary pulmonary hypertension. Circulation.

[35] Richelle, A. *et al.* What does your cell really do? Model-based assessment of mammalian cells metabolic functionalities using omics data. bioRxiv. 2020:2020.04.26.057943. 10.1016/j.crmeth.2021.100040 PMC8577426 34761247

[36] Elinoff JM (2018). ()2018 Challenges in pulmonary hypertension: controversies in treating the tip of the iceberg. A joint National Institutes of Health Clinical Center and Pulmonary Hypertension Association Symposium report. Am. J. Respir. Crit. Care. Med..

[37] Nemkov T, Hansen KC, D'Alessandro A (2017). A three-minute method for high-throughput quantitative metabolomics and quantitative tracing experiments of central carbon and nitrogen pathways. Rapid. Commun. Mass Spectrom..

[38] Catalin, E., & Doneanu W. C. Application note 2011, 720004042en. UPLC/MS Monitoring of Water-Soluble Vitamin Bs in Cell Culture Media in Minutes. https://www.waters.com/waters/library.htm?locale=en_US&lid=134636355) (2020).

[39] Johnsen LG, Skou PB, Khakimov B, Bro R (2017). Gas chromatography - mass spectrometry data processing made easy. J. Chromatogr. A..

[40] Stekhoven DJ, Bühlmann P (2012). MissForest–non-parametric missing value imputation for mixed-type data. Bioinformatics.

[41] Schellenberger J (2011). Quantitative prediction of cellular metabolism with constraint-based models: the COBRA Toolbox v20. Nat. Protoc..

